# Enpp2 Expression by Dendritic Cells Is a Key Regulator in Migration

**DOI:** 10.3390/biomedicines9111727

**Published:** 2021-11-19

**Authors:** Jun-Ho Lee, So-Yeon Choi, Soo-Yeoun Park, Nam-Chul Jung, Kyung-Eun Noh, Ji-Hee Nam, Ji-Soo Oh, Hyun-Ji Choi, Ji-Su Jang, Ji-Young Yoo, Jie-Young Song, Han Geuk Seo, Dae-Seog Lim

**Affiliations:** 1 Department of Biotechnology, CHA University, 335 Pangyo-ro, Bundang-gu, Seongnam 13488, Gyeonggi-do, Korea; jhlee@pharosvaccine.com (J.-H.L.); csy5900@naver.com (S.-Y.C.); sooyeoun92@naver.com (S.-Y.P.); nge6@naver.com (K.-E.N.); wl08gml03@naver.com (J.-H.N.); jisoo821@naver.com (J.-S.O.); hjc1991@naver.com (H.-J.C.); danio0820@naver.com (J.-S.J.); joy.cc6000@gmail.com (J.-Y.Y.); 2 Pharos Vaccine Inc., 14 Galmachiro 288 bun-gil, Jungwon-gu, Seongnam 13201, Gyeonggi-do, Korea; ncjung@pharosvaccine.com; 3 Department of Radiation Cancer Sciences, Korea Institute of Radiological and Medical Sciences, 75 Nowon-ro, Nowon-gu, Seoul 01812, Korea; immu@kcch.re.kr; 4 Department of Food Science and Biotechnology of Animal Products, Sanghuh College of Life Sciences, Konkuk University, 120 Neungdong-ro, Gwangjin-gu, Seoul 05029, Korea; hgs-eo@konkuk.ac.kr

**Keywords:** Enpp2, dendritic cells, migration

## Abstract

Enpp2 is an enzyme that catalyzes the conversion of lysophosphatidylcholine (LPC) to lysophosphatidic acid (LPA), which exhibits a wide variety of biological functions. Here, we examined the biological effects of Enpp2 on dendritic cells (DCs), which are specialized antigen-presenting cells (APCs) characterized by their ability to migrate into secondary lymphoid organs and activate naïve T-cells. DCs were generated from bone marrow progenitors obtained from C57BL/6 mice. Enpp2 levels in DCs were regulated using small interfering (si)RNA or recombinant Enpp2. Expression of Enpp2 in LPS-stimulated mature (m)DCs was high, however, knocking down Enpp2 inhibited mDC function. In addition, the migratory capacity of mDCs increased after treatment with rmEnpp2; this phenomenon was mediated via the RhoA-mediated signaling pathway. Enpp2-treated mDCs showed a markedly increased capacity to migrate to lymph nodes in vivo. These findings strongly suggest that Enpp2 is necessary for mDC migration capacity, thereby increasing our understanding of DC biology. We postulate that regulating Enpp2 improves DC migration to lymph nodes, thus improving the effectiveness of cancer vaccines based on DC.

## 1. Introduction

Migration of immune cells is a biological process fundamental to normal physiology [1,2]. This process increases the chance that lymphocytes will encounter an antigen and trigger an effective inflammatory response against the pathogen from which the antigen is derived. Autoimmune disorders and unexpected excessive immune responses are associated with abnormal immune cell migration and erroneous recognition of shed autoantigens by antigen-presenting cells (APCs) [3,4]. Some studies provided strong support for this idea, and pharmacological inhibitors have been developed to block immune cell migration [5,6,7]. Some of these inhibitors are highly effective treatments for certain disease conditions [8,9,10]. By contrast, biological processes, such as anti-cancer immunity, require a fully functional and active immune response. The presence of active APCs in patients with impaired immune function is extremely important for successful treatment [11]. 

Dendritic cells (DCs) are professional APCs. As such, they induce cellular and humoral immune responses against cancer cells by expressing cell-derived antigens to T-cells via major histocompatibility complex (MHC) class I/II complexes. To generate DC vaccines, immature (im)DCs are exposed to antigens, then converted to fully mature (m)DCs. The mDCs signal T-cells to present antigen-specific peptides on MHC class I/II complexes; express high levels of costimulatory molecules, such as B7-1 (CD80), B7-2 (CD86), and CD40; and secrete large amounts of pro-inflammatory cytokines. Moreover, during the maturation process, DCs show increased migration activity and homing to lymph nodes (LNs) [12]. Migration of DCs to secondary lymphoid organs and their interactions with antigen-specific T-cells are prerequisites for induction of a primary immune response [13,14]. Normally, DC vaccines are injected subcutaneously; the cells then migrate to draining LNs through the lymphatic vessels. However, the percentage of injected DCs that migrate to the LNs is very low. Previous studies reported that the migratory properties of bone marrow-derived DCs (BMDCs) are increased by LPS/TLR4-signaling, induced via a rapid increase in the concentration of free cytosolic calcium ions [15]. 

G protein-coupled receptors (GPCRs), which are seven transmembrane receptors, are activated by a variety of factors ranging from small amines to hormones and chemokines. The lysophospholipids sphingosine 1-phosphate (S1P) and lysophosphatidic acid (LPA) signal through GPCRs, which couple to multiple G-proteins and their effectors [16]. Similar to cytokines, they are locally-acting, short-lived molecules that signal through specific cell surface receptors [17]. Under physiological conditions, S1P regulates lymphocyte egress from lymphoid tissues, whereas LPA regulates lymphocyte ingress into and migration within LNs [17]. LPA are membrane-derived phospholipids that arise from homeostatic lipid metabolism, or as a response to stimulus-induced cellular activation. The majority of circulating LPA originates from the enzymatic activity of ectonucleotide pyrophosphatase/phosphodiesterase 2 (Enpp2; also called autotaxin (ATX)), which is a plasma lysophospholipase D (Lyso-PLD); indeed, mice heterozygous for the knockout allele for Enpp2 show a 50% reduction in LPA levels [18,19]. Enpp2 is a member of the ectonucleotide pyrophosphatase/phosphodiesterase family of enzymes that hydrolyze the phosphodiesterase bonds of various nucleotides and derivatives. Originally, Enpp2 was isolated as an autocrine motility factor from the supernatant of melanoma cells. Since then, increased expression of Enpp2 has been detected in a variety of cancers. In general, the effect of LPA on a particular cell type will depend ultimately on the local concentration (determined by Enpp2 activity), the relative levels of agonists and antagonists, the relative abundance of different receptor subtypes, and the signals transduced by each LPA receptor to its associated G protein; the latter controls a large number of cellular functions. Ligand binding to these receptors results in activation of multiple signaling pathways with various downstream physiological and pathological effects [17]. For example, RhoA belongs to the family of small GTPases, and is the major downstream signaling molecule of LPA-LPA receptor signaling; Gα12/13 induces RhoA-dependent cytoskeletal remodeling, cell migration, and invasion in concert with the Gαi-mediated Rac activation pathway. 

In this study, we examined the biological effect of Enpp2 on DCs, and show that Enpp2 enhances migration of DCs via the RhoA signaling cascade, thereby driving increased migration to LNs in vivo.

## 2. Materials and Methods

### 2.1. Ethics Approval

All protocols involving the use of animals were approved by the Institutional Animal Care and Use Committee of CHA University (Project IACUC150051, 3 June 2015), and all experiments were carried out in accordance with these approved protocols.

### 2.2. Mice

Male C57BL/6 mice (6–8 wk of age, 14–16 g) were purchased from Orient Bio (Seongnam, Korea). All mice were housed in a temperature- and humidity-controlled room under a 12-h light/dark cycle.

### 2.3. Generation of DCs

Mouse DCs were generated from bone marrow progenitor cells obtained from C57BL/6 mice [20]. Single bone marrow cells were obtained and then washed with RPMI 1640 medium, followed by culture at 37 °C/5% CO_2_ in RPMI 1640 (Lonza, Basel, Switzerland) containing 10% fetal bovine serum (FBS) (Corning, Glendale, NY, USA), 20 ng/mL recombinant mouse (rm) granulocyte-macrophage colony-stimulating factor (GM-CSF) (JWCreaGene, Gyeonggi, Korea), 20 ng/mL rm Interleukin (IL)-4 (JWCreaGene), and 50 nM mercaptoethanol (2-ME) (Invitrogen, Waltham, MA, USA). On day 2, non-adherent cells were washed and re-fed with the same culture medium. On day 4, half of the culture medium was replaced with the same culture medium. On day 6, non-adherent cells were collected and incubated for 24 h with 1 μg/mL lipopolysaccharide (LPS) (Sigma-Aldrich, St. Louis, MO, USA) and 10 μg/mL keyhole limpet hemocyanin (KLH) (Sigma). At the same time, some cultures were treated with 10 ng/mL rmEnpp2 or 1 μM Enpp2 inhibitor (PF8380). Normal human DCs (CC-2701; Lonza) were cultured with serum free media (X-VIVO15; Lonza), 20 ng/mL recombinant human GM-CSF, and IL-4 (R&D system), and differentiated to mature DCs with the addition of 1 μg/mL LPS (Sigma) and 1 μg/mL prostaglandin E2 (PGE2) (Sigma). 

### 2.4. RNA Interference

DCs were plated into each well of a 6-well plate (SPL Life Sciences, Gyeonggi, Korea), and transfected with small interfering RNA (siRNA) duplexes using the Lipofectamine 3000 transfection reagent (Invitrogen), according to the manufacturer’s instructions, to deliver the siRNAs (Enpp2 siRNAs; sense: 5′-GGGUCUUGGUGAAGAAAUAdTdT-3′; antisense: 5′-UAUUUCUUCACCAAGACCCdTdT-3′ and scramble-siRNA) to Bioneer (Daejeon, Korea). On day 6, the cells were cultured for 4 h with the transfection mixture (Lipofectamine-100 nM Enpp2 siRNA mixture) in an OptiMEM medium (Invitrogen), before being stimulated with 1 μg/mL LPS for 24 h.

### 2.5. Flow Cytometry Analysis

Cells were incubated with antibodies on ice for 20 min, and then washed with PBS containing 1% BSA and 0.05% NaN_3_. For surface phenotype analysis, cells were stained with the following fluorescence-conjugated monoclonal antibodies: anti-CD11c (HL3), anti-H-2Db (KH95), anti-I-Ab (AF6-120.1), anti-CD80 (16-10A1), anti-CD86 (GL1), anti-CD40 (3/23), anti-CD54 (3E2), anti-CD14 (rmC5-3), anti-CD4 (RM4-5), and anti-CD8α (53-6.7). Isotype controls consisted of cells labeled with rat or hamster Ig. All antibodies were purchased from BD Biosciences (San Diego, CA, USA). To measure viability, cells were stained with propidium iodide (BD Biosciences). To measure antigen-uptake capacity, DCs were incubated for 1 h with 1 μg/mL FITC-dextran, and then washed in PBS twice. Finally, cells were examined using a FACSCalibur flow cytometer (BD Biosciences). Data were analyzed using FlowJo software (TreeStar^TM^ Inc., San Carlos, CA, USA).

### 2.6. Cytokine Measurement

Cytokine (IL-1β, IL-6, IL-12p70, and TNF-α) levels were measured using a sandwich enzyme-linked immunosorbent assay (ELISA) kit (BD Biosciences), according to the manufacturer’s instructions. Briefly, DCs were cultured for 24 h in the absence of GM-CSF and IL-4. Next, the culture supernatant was harvested, and cytokine levels were measured in the ELISA. To verify induction of helper T-cells, (Th)1 and Th17, immune responses by DCs, CD3-positive T-cells from the spleen were cultured for 72 h with DCs in 6-well plates (2 × 10^5^ cells/well, DC:T ratio = 1:10). The culture supernatant was harvested and Interferon gamma (IFN-γ), IL-17A, IL-4, and IL-10 production was measured in an ELISA kit (BD Biosciences). All samples were assayed in triplicate.

### 2.7. Western Blotting

To detect intracellular signaling in DCs, recombinant Enpp2-treated DCs, Enpp2 siRNA-transfected DCs (siEnppP2 DCs), Enpp2 inhibitor (PF8380)-treated DCs, imDCs, and mDCs were lysed using the Proprep^TM^ protein extraction kit (Intron Biotechnology, Gyeonggi, Korea) in the presence of a serine/threonine phosphatase inhibitor cocktail (Sigma-Aldrich). Equal amounts of protein (10 μg) were separated on SDS-PAGE gels, and transferred to PVDF membranes. The membranes were blocked with 5% (w/w) skim milk in TBST, and then incubated with primary antibodies specific for pErk1/2, Erk1/2, p-JNK, JNK2, pp38, p38, NF-κBp65, or GAPDH (all diluted 1:1000; Cell Signaling Technology, Danvers, MA, USA). The membranes were then incubated with an HRP-conjugated secondary antibody (diluted 1:2000; Cell Signaling Technology). To detect Enpp2, a culture supernatant was concentrated by ultracentrifugation. Proteins in samples were separated and transferred to PVDF membranes prior to detection with an anti-ENPP2 antibody (Cayman Chemical, Ann Arbor, MI, USA) and an HRP-conjugated anti-rabbit antibody (Abcam, Cambridge, MA, UK). The membranes were exposed to ECL reagents (Thermo Scientific), and signals were detected using a Luminescent image analyzer (LAS-4000; Fujifilm, Tokyo, Japan).

### 2.8. Quantitative qRT-PCR 

Total RNA was extracted using Trizol reagent (Life Technologies, Carlsbad, CA, USA). The prepared RNA was subjected to DNase digestion, and the concentration was measured using an Agilent 2100 Bioanalyzer (Agilent Technologies, Palo Alto, CA, USA). Total RNA was reverse transcribed using the SensiFAST^TM^ cDNA Synthesis kit (Bioline^TM^, Annapolis, MD, USA), and cDNA samples were analyzed by quantitative RT-PCR (qRT-PCR) using specific primers and the SensiFAST^TM^ SYBR^®^ High-ROX kit (Bioline^TM^). The primer sequences are listed in Supplementary Table S1. PCR was conducted using a Bio-Rad CFX96^TM^ Real-Time PCR detection system (Bio-Rad Laboratories, Inc., Hercules, CA, USA). GAPDH was amplified as an internal control. Relative expression was calculated using the ΔΔ*Ct* method, and expressed as -fold changes using the formula 2^−ΔΔ*Ct*^. All experiments were run in triplicate.

### 2.9. Enpp2 Activity Assay

DCs were cultured for 24 h in RPMI 1640 containing 2% FBS. Cell culture supernatants were collected and tested in a commercial Enpp2 activity assay (Echelon, K-4100). Enpp2 activity in the medium was analyzed using a fluorescent Enpp2 substrate, FS-3, according to the manufacturer’s protocol [21]. Briefly, 50 μL of reaction solution were added to 50 μL of conditioned medium in a 96-well plate. FS-3 is an LPC analogue that is conjugated to both a fluorophore and a quencher. Once Enpp2 cleaves FS-3, the fluorophore is liberated from the quencher, resulting in increased fluorescence. Fluorescence intensity was measured in a multi-mode plate reader Synergy H1 (BioTek Instruments, Inc., Winooski, VT, USA) at an excitation wavelength of 485 nm and an emission wavelength of 528 nm. Readings were taken every 2 min for a period of 2 h. 

### 2.10. Migration Assay and In Vivo Tracking

To determine whether Enpp2-mediates migration of DCs siEnpp2-, Enpp2 inhibitor (PF8380)-, or rm Enpp2-treated DCs (5 × 10^5^ cells/500 μL) were added to the upper chamber of a Transwell (in serum-free medium), and 100 ng/mL CCL19 was added to the lower chamber. After 1 h of incubation at 37 °C/5% CO_2_, the migrated DCs were harvested from the lower chamber, and counted in a FACSCalibur cytometer (1 min per count). For visualization and imaging, DCs were labeled with carboxyfluorescein succinimidyl ester (CFSE) (Invitrogen) or IRDye^®^ 800CW NHS Ester NIR-dye (Li-COR Biosciences, Lincoln, NE, USA), respectively. Cells (5 × 10^4^ rmEnpp2-treated DCs or non-treated DCs) were injected into the footpad of C57BL/6 mice. Imaging of DC-injected mice was performed using an imaging system comprising an excitation light source and an image capture (NIR imaging system) system; images were captured every 24 h for a period of three days. At three days post-injection of CFSE-labeled DCs, mice were sacrificed and popliteal LNs were harvested. Total cells were isolated from the popliteal LNs, and CFSE/CD11c-positive cells were analyzed in a FACSCalibur cytometer. For some samples, CFSE signals in LNs were visualized under a confocal microscope (Carl Zeiss, Thornwood, NY, USA). 

### 2.11. Statistical Analysis

Statistical analysis was performed using GraphPad software (GraphPad Prism v7.0; GraphPad Software, San Diego, CA, USA). Data were analyzed by using a paired t-test or two-way ANOVA followed by the Newman–Keuls test. Results are expressed as the mean ± SEM. A *p*-value < 0.05 was considered significant.

## 3. Results

### 3.1. mDCs Secrete More Enpp2 Protein Than imDCs 

**Figure 1:**
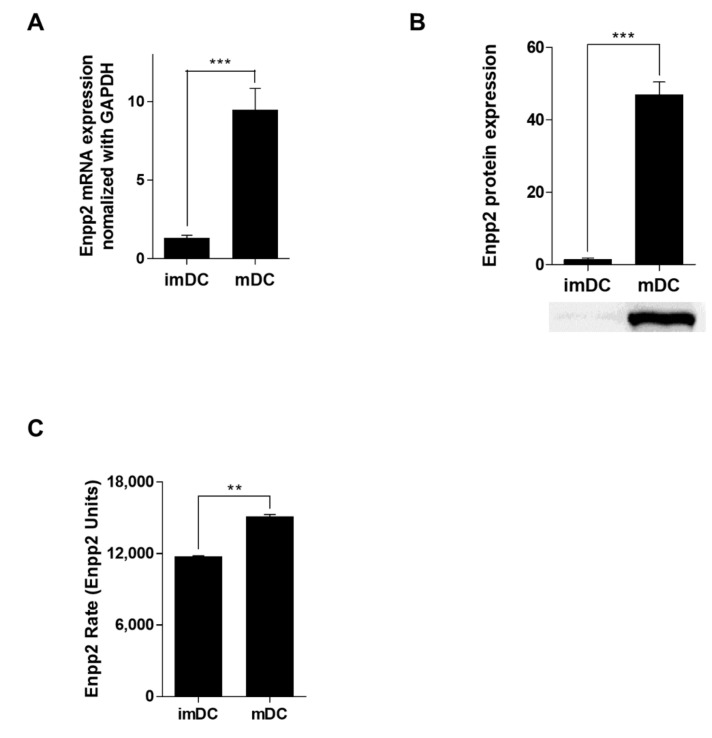
mDCs secrete high levels of Enpp2. (**A**) DC subsets were cultured and Enpp2 expression measured by qRT-PCR. Values were normalized to GAPDH expression. Data are expressed as the mean ± SEM (*n* = 5 independent DC preparations) of duplicate experiments. (**B**) Secreted Enpp2 protein in concentrated (20-fold) conditioned low serum medium from DCs was detected by Western blotting. The bar graphs show data derived from densitometric analysis of western blot data. Values are expressed as the mean ± SEM (*n* = 3 independent experiments). (**C**) A fluorescence assay of Enpp2 activity. Enzyme activity in conditioned medium was measured for over 2 h. The bar graph shows the Enpp2 activity rates, expressed as the mean ± SEM (*n* = 3 independent DC preparations). ** *p* < 0.01 and *** p < 0.001, compared with immature (im)DCs.

Previously, we conducted microarray mRNA profiling, and found that mDCs express high levels of mRNA encoding Enpp2 [22]. First, we showed that DCs generated from bone marrow progenitor cells expressed CD11c and MHC II. mDCs showed higher expression of costimulatory molecules CD40, CD54, CD80, and CD86 than imDCs (Supplementary Figure S1A). In addition, mDCs secreted more pro-inflammatory cytokines (IL-1β, IL-6, IL-12p70, and TNF-α) than imDCs (Supplementary Figure S1B). Phagocytic analysis revealed that phagocytic activity by mDCs was markedly lower than that of imDCs (Supplementary Figure S1C). The results of the Transwell assay indicated, that during the maturation process, mDCs showed increased migration activity (Supplementary Figure S1D, Supplementary Table S2). Supplementary Figure S1E was confirmed by Western blot analyses showing that mDCs expressed higher levels of RhoA protein than imDCs. To examine the effect of DCs on T-cells, we conducted co-culture experiments; the results showed that mDCs induced T-cell proliferation more efficiently than imDCs (Supplementary Figure S1F). T-cells co-cultured with mDCs showed a marked increase in production of IFN-γ (a Th1 cytokine) and IL-17A (a Th17 cytokine) (Supplementary Figure S1G,H). Next, we confirmed that mDCs expressed higher levels of Enpp2 mRNA than imDCs (Figure 1A); they also secreted more Enpp2 into the culture supernatant than imDCs (Figure 1B). Since tiny differences in Enpp2 concentrations in different biological samples can have very different enzymatic activities, we measured Enpp2 activity in DC-conditioned in medium using a fluorescent substrate, FS-3. The rate of FS-3 cleavage (expressed as relative fluorescence units) in cell culture supernatant from mDCs was much higher (i.e., greater enzymatic activity) than that in supernatant from imDCs (Figure 1C). 

### 3.2. Enpp2 Does Not Affect DC Maturation, But Does Affect Immunogenicity

**Figure 2:**
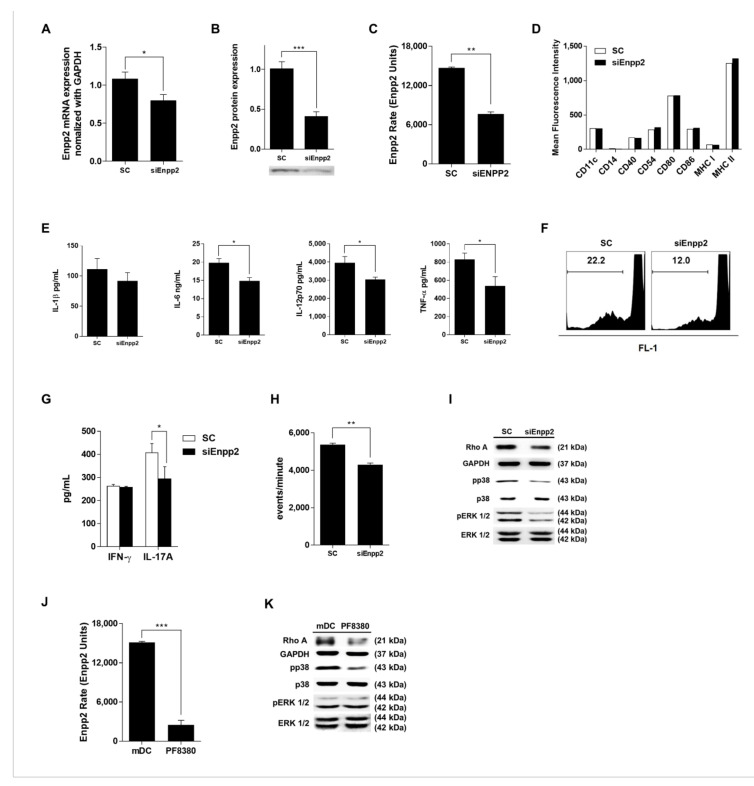
Enpp2 knockdown in mDCs affects their immunogenicity. (**A**) Expression of Enpp2 mRNA in DC subsets (scramble siRNA-treated mDCs; SC, and Enpp2 siRNA-treated mDCs; siEnpp2). Enpp2 expression was measured by qRT-PCR. Values were normalized against GAPDH expression. The bar graph shows the Enpp2 expression relative to levels in imDCs. Data are presented as mean ± SEM (*n* = 5 independent DC preparations). (**B**) Levels of secreted Enpp2 protein in concentrated (20-fold) conditioned low serum medium from SC or siEnpp2 were detected by Western blotting. Data in the bar graphs were obtained by densitometric analysis of Western blots. All data are expressed as the mean ± SEM (*n* = 3 independent experiments). (**C**) Fluorescence assay of Enpp2 activity. Enzyme activity in conditioned medium was measured for over 2 h. The bar graph shows the Enpp2 activity rates, expressed as the mean ± SEM (*n* = 3 independent DC preparations). (**D**) SC and siEnpp2 were stained with the indicated fluorescent-labeled antibodies and analyzed by flow cytometry. The bar graphs show the mean fluorescence intensity (MFI). (**E**) Pro-inflammatory cytokines in culture supernatants from DCs were analyzed by ELISA. Data are expressed as the mean ± SEM (*n* = 5 independent DC preparations) of duplicate experiments. (**F**) Each DC subset was co-cultured for 72 h with CD3^+^ T cells at a ratio of 1:10. The CD3^+^ T-cells isolated from splenocytes of naïve C57BL/6 mice were stained with CFSE and co-cultured with DCs. (**G**) Cytokine levels in the supernatants after 72 h of co-culture were measured by ELISA. Data are expressed as mean ± SEM (*n* = 3 independent co-culture preparations) of duplicate experiments. (**H**) Migration assay. The number of migrating DCs harvested from the lower Transwell chamber was counted by flow cytometry. The bar graph shows the events/minute, expressed as the mean ± SEM (*n* = 3 independent DC preparations). (**I**) To detect the indicated proteins, DC lysates were subjected to SDS-PAGE and immunoblotting. Data shown are representatives of three independent experiments. Western blot analysis of RhoA, p38, Erk1/2 expression. (**J**) Fluorescence assay of Enpp2 activity. Enzyme activity in conditioned medium was measured for over 2 h. The bar graph shows the Enpp2 activity rates, expressed as the mean ± SEM (*n* = 3 independent DC preparations). (**K**) To detect the indicated proteins, DC lysates were subjected to SDS-PAGE and immunoblotting. Data shown are representatives of three independent experiments. Western blot analysis of RhoA, p38, Erk1/2 expression. * p < 0.05, ** p < 0.01, and *** p < 0.001, compared with SC.

To investigate the effects of Enpp2 on DCs, we transfected cells with siRNA targeting Enpp2 (Enpp2-siRNA). The results confirmed downregulated expression of Enpp2 mRNA and protein (Figure 2A,B). Moreover, mDCs transfected with Enpp2-siRNA (siEnpp2) showed a marked reduction in Enpp2 activity (Figure 2C). However, knockdown did not affect expression of cell surface molecules (Figure 2D). Interestingly, knocking down Enpp2 reduced the immunogenicity of mDCs, resulting in decreased cytokine production (Figure 2E). To test whether siEnpp2 lose their ability to stimulate T-cells, we co-cultured DCs with T-cells, and found decreased T-cell proliferation in the presence of siEnpp2 (Figure 2F). In addition, there was a marked decrease in the Th1 T-cell and Th17 T-cell populations when co-cultured with siEnpp2. Moreover, siEnpp2 reduced secretion of IL-17A by T-cells. There was no significant difference in secretion of IFN-γ (Figure 2G), and IL-4 and IL-10 were not detected. Next, to reveal the role of Enpp2 in DC migration, we conducted a Transwell migration assay. The migration activity of siEnpp2 was significantly lower than SC (Figure 2H, Supplementary Table S3). Furthermore, Western blot analysis revealed that expression of RhoA, pp38, and pERK 1/2 by siEnpp2 was lower than that in SC (Figure 2I). However, there was no difference in JNK and NFkB signaling pathways. Next, as FBS contains Enpp2 components, we treated mDCs with an Enpp2 inhibitor (PF8380) to inactivate secreted Enpp2. Enpp2 secreted by DCs treated with PF8380 showed markedly reduced activity (Figure 2J). Western blot data confirmed the above siEnpp2 results: pharmacologic inhibition of Enpp2 attenuated the RhoA and pp38 signaling pathways (Figure 2K). Taken together, these results suggest that Enpp2 affects the immunogenicity of mDCs via the RhoA and pp38 signaling pathways.

### 3.3. Enpp2 Increases the Immunogenicity of mDCs by Stimulating Maturation and Migration

**Figure 3:**
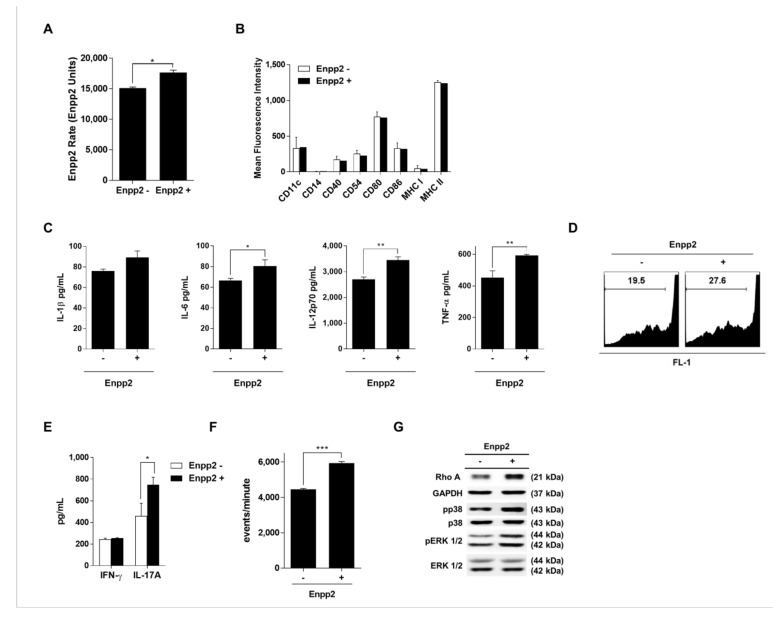
Enpp2 increases the immunogenicity of mDCs. (**A**) Fluorescence assay of Enpp2 activity. Enzyme activity in conditioned medium was measured over 2 h. The bar graph shows the Enpp2 activity rates, expressed as the mean ± SEM (*n* = 3 independent DC preparations). (**B**) DC subsets (mDC; Enpp2- and recombinant Enpp2 protein treated mDCs; Enpp2+) were stained with the indicated fluorescent-labeled antibodies and analyzed by flow cytometry. The bar graphs show the MFI, expressed as the mean ± SEM (*n* = 3 independent DC preparations). (**C**) Pro-inflammatory cytokines in culture supernatants from DCs were analyzed by ELISA. Data are expressed as the mean ± SEM (*n* = 3 independent DC preparations) of duplicate experiments. (**D**) Each DC subset was co-cultured for 72 h with CFSE-labeled CD3^+^ T-cells to measure T-cell proliferation. (**E**) Cytokine levels in the supernatants after 72 h of co-culture were measured by ELISA. Data are expressed as mean ± SEM (*n* = 3 independent co-culture preparations) of duplicate experiments. (**F**) Migration assay. The number of migrating DCs harvested from the lower Transwell chamber was counted by flow cytometry. The bar graph shows the events/minute, expressed as the mean ± SEM (*n* = 3 independent DC preparations). (**G**) Western blot analysis of RhoA, p38, Erk1/2 expression. Data are representative of three independent experiments. * p < 0.05, ** p < 0.01, and *** p < 0.001, compared with Enpp2-.

To further investigate the effect of Enpp2 on mDCs, we exposed maturing mDCs to rmEnpp2. Although Enpp2 activity in maturing mDCs increased slightly (Figure 3A), there was no difference in expression of surface marker proteins by treated and untreated cells (Figure 3B). However, there was a significant increase in cytokine production and T-cell proliferation in the presence of rmEnpp2 (Figure 3C,D), along with a more pronounced Th17 (IL-17-secreting) profile. There were no significant differences in secretion of IFN-γ (Figure 3E). In addition, there was a marked increase in migration activity and expression of RhoA and pp38 protein by mDCs in the presence of Enpp2 (Figure 3F–G, Supplementary Table S4). 

**Figure 4:**
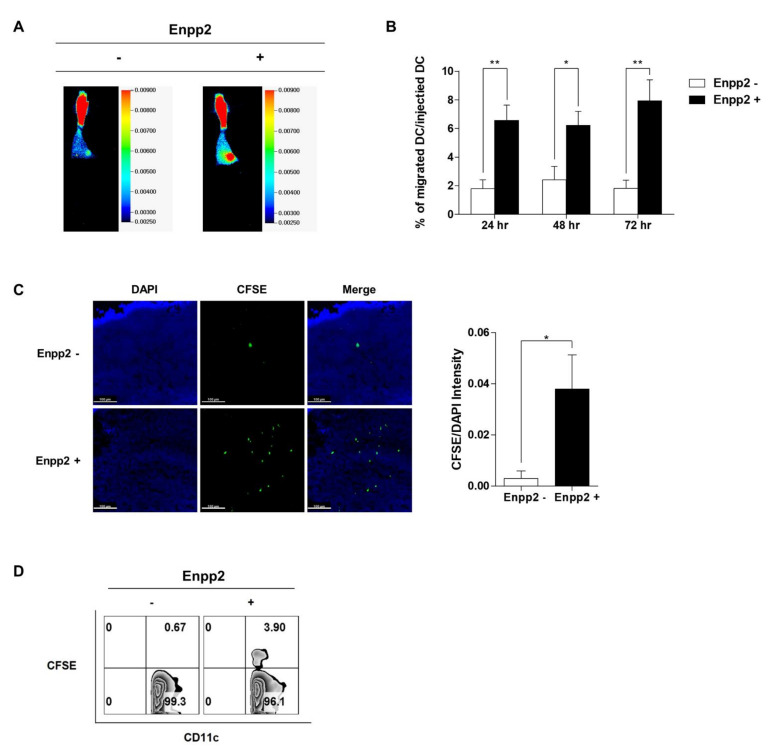
Enpp2 increases the homing ability of mDCs in vivo. DCs were injected subcutaneously into the right hind leg footpad of C57BL/6 mice, and imaged at 24, 48, and 72. (**A**) DCs (5 × 10^4^ cells/50 μL) were injected subcutaneously into mouse (*n* = 5 mice for each group) footpads to monitor cell migration to the popliteal lymph nodes. Imaging was performed using the near-infrared 800 nm channel, exciting at a wavelength of 778 nm and detecting at 794 nm. Representative optical images acquired on day 3 post-injection. (**B**) The bar graphs show that fluorescence intensity of DCs migrated to the popliteal lymph nodes (total fluorescence intensity is shown for comparison). Data are expressed as the mean ± SEM (*n* = 5 independent mice). (**C**) CFSE (green)-labeled DCs were injected subcutaneously into mouse (*n* = 3 mice per group) footpads and confocal micrographs of the popliteal lymph nodes were acquired. Nuclei were stained with DAPI (blue). All images were taken using the same microscope settings and representative images are shown. The bar graphs show the CFSE/DAPI intensity. Scale bar, 100 μm. (**D**) Cells were isolated from the popliteal lymph nodes of each group of mice and then stained with an anti-CD11c antibody prior to flow cytometry analysis. The percentage of CFSE-labeled DCs is indicated on the plots. Data are representative of three independent experiments. * p < 0.05 and ** p < 0.01 compared with Enpp2-.

Finally, to study the effect of Enpp2 on migration of mDCs, cells were labeled with IRDye^®^ 800CW or with CFSE, and then injected into the right footpad of C57BL/6 mice. The signals generated by the labeled DCs were monitored using an in vivo imaging system. NIR (near-infrared) signals appeared in the popliteal LNs at 24 h post-injection of IRDye^®^ 800CW labeled DCs; this signal intensified up until 72 h post-injection (Figure 4A,B). Migration of rmEnpp2-treated cells (Enpp2+) was markedly superior to that of untreated cells (Enpp−). Ex vivo imaging of popliteal LNs dissected from both limbs confirmed that the observed CFSE signals were generated within the draining LNs on the side ipsilateral to the DCs injection site (Figure 4C; Supplementary Video S1 and S2). Representative flow cytometry data showed that the vast majority of CFSE-positive cells within the gated area were CD11c-positive. However, the percentage of CFSE/CD11c-positive rmEnpp2-treated mDCs in the popliteal LNs of injected mice was 5-fold higher than that of non-treated mDCs (3.9% vs. 0.67%, respectively; Figure 4D). These results suggest that Enpp2 increases the migration capacity of mDCs in vivo.

### 3.4. Human mDCs Secrete More Enpp2 Protein Than imDCs

**Figure 5:**
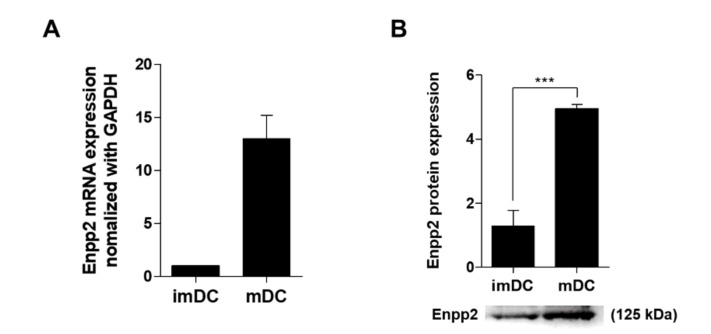
Human mDCs secrete high levels of Enpp2. (**A**) Human DC subsets (imDC and mDC) were cultured, and Enpp2 expression measured by qRT-PCR. Values were normalized to GAPDH expression. Data are expressed as the mean ± SEM (*n* = 5 independent DC preparations) of duplicate experiments. (**B**) Levels of secreted Enpp2 protein in concentrated (20-fold) conditioned serum free media (SFM) from DCs was detected by western blotting. The bar graphs show data derived from densitometric analysis of western blot data. Values are expressed as the mean ± SEM (*n* = 3 independent experiments). *** p < 0.001 compared with imDC.

In addition to the features of Enpp2 expression in mouse DCs, Enpp2 has also been explored in human DCs. Since the human serum contains Enpp2 (Supplementary Figure S2), serum free media (SFM) was used for generation of DCs. Interestingly, similar to the results from mouse DCs, the levels of Enpp2 mRNA and protein expression in human mDCs was higher than in human imDCs (Figure 5A,B).

## 4. Discussion

The basic principle underlying DC vaccines is very simple [23]; however, there are many immunological determinants to consider during manufacture. These include the method used for tumor antigen loading, cocktails used for DC maturation, routes of administration, and vaccine dosage and scheduling [24]. The number of DCs that can be isolated from a patient is very low; therefore, even when administered in small doses, it is of utmost important that DCs migrate efficiently to the T-cell area within the LNs.

In a previous study, we examined the gene expression profile from each subset of DCs and identified the *Enpp2* gene as being highly expressed by mDCs. Since Enpp2-deficient mice died at embryonic day 9.5 with profound vascular defects [19], we generated mDCs showing downregulated expression of Enpp2. Although these cells showed normal expression of immunophenotypic markers, their function (cytokine release, T-cell activation, and migratory capacity) was inhibited (Figure 2). In Figure 2E, siEnpp2 decreased cytokine production, with the exception of IL-1b. There may be no change in NFkB signaling, the greatest effect on the expression of IL-1, due to the resulting siEnpp2 [25]. In particular, knockdown of Enpp2 reduced migratory capacity in vitro (Figure 2H). In addition, knockdown reduced expression of RhoA protein, suggesting that the reduced migratory capacity of DCs is regulated by a RhoA-mediated signaling pathway (Figure 2I). 

Taken together, these results indicate that Enpp2 plays an important role in the function of mDCs, especially migration. Enpp2 is an ectoenzyme expressed and secreted by many cell types, and plays roles in blood vessel formation, cancer cell migration, insulin resistance in muscle cells, and homing and inflammatory responses by lymphocytes [10,18,26,27,28,29,30]. Here, we exposed cultured DCs to recombinant Enpp2 protein during the maturation process. In contrast to siEnpp2, rmEnpp2-treated mDCs showed improved cytokine release, T-cell activating ability, migratory capacity in vitro (Figure 3) and in vivo (Figure 4). We also found that the migratory capacity of DCs and expression of RhoA protein increased and decreased as the expression of Enpp2 increased and decreased. These results are consistent with those reported by previous studies showing that activation of the Gα12/13 subunit activates RhoA/ROCK, which is associated with DC migration [31]. Additionally, RhoA is important for the presentation of pathogen-derived antigen and the formation of an immunological synapse between DCs and antigen-specific T-cells, which is a prerequisite for induction of adaptive T-cell responses [32]. Furthermore, the experiments reported in Figure 2 using additional siRNAs for Enpp2 will make the data more robust and reliable.

Enpp2 expression is induced at inflammatory sites, which leads to local production of LPA. LPA is stored within cells, and may accumulate in extracellular fluids as an inflammatory exudate, in which it is suggested to play an immunoregulatory role [33]. Aberrant production and/or metabolism of these lysophospholipids results in dysregulated distribution of immune cells, and induction of various types of inflammatory response in vivo [17]. Although there are some studies demonstrating that LPA affects DC activation [34,35], since LPA is rapidly eliminated from the circulation [36] and has a short half-life in the blood [17,37], we conducted a study using Enpp2. In the LNs, stromal cells produce Enpp2, and the Enpp2-LPA axis regulates T-cell mobility dependent on the Rho-ROCK-myosin II pathway [38,39]. Lysophospholipid receptors that couple to RhoA signaling are involved in various pathophysiological conditions, suggesting that these receptors are important targets for disease therapy [16]. Therefore, understanding the specificity of lysophospholipid receptor coupling to RhoA activation, and the associated lipid signaling pathways, may provide additional strategies for treatment of various types of inflammatory diseases.

With respect to signaling pathways, we found that pp38 was expressed at low levels in siEnpp2- and PF8380-treated mDCs. This is consistent with a study showing that activation of p38 MAPK protein is required for differentiation into mature dendritic cells [40]. In addition, the results of a previous study confirm that activation of the RhoA protein is dependent on pp38 [41].

Some studies [42] show that Enpp2 expressed in LPS-treated human moDCs modulates the immune response, but no studies have examined migration. Various chemokines, receptors, and molecules affect migration of DCs to secondary lymphoid organs. Chemokines are the most well-known activators of immune cell migration; a good example is the Mip3β/C-C chemokine receptor (CCR7) axis. Moreover, DCs express CCR7 during the maturation process, which increases migration activity and homing to LNs [12]. CCR7 is a key regulator of lymphocyte trafficking to secondary lymphoid organs [43]. Chemokine C-C motif ligand (CCL)19 and CCL21 are the two ligands for CCR7; in mice, these ligands are expressed in the afferent lymphatic vessels, and in the LN paracortex and subcapsular sinus. Bone marrow-derived DCs (BMDCs) have been used to demonstrate the role played by CCR7 in DCs. CCR7 is a very important factor for DC migration; however, we did not find a significant change of expression in siEnpp2- and rmEnpp2-treated mDCs (data not shown). It can be seen that DC migration regulation by Enpp2 is CCR7 independent, and affects RhoA signal. 

To summarize, the results presented herein confirm that Enpp2, a protein involved in cell migration, is expressed by mDCs. In addition, analyses of Enpp2 knockdown or Enpp2-treated mDCs revealed that Enpp2 is a key factor that regulates the biological function of DCs. Thus, we suggest that regulating Enpp2 may improve DC migration to LNs, thereby improving the efficacy of DC-based cancer vaccines.

## Data Availability

All data generated or analyzed during this study are included in this published article [and its Supplementary Materials].

## Supplementary Materials

The following are available online at https://www.mdpi.com/article/10.3390/biomedicines9111727/s1, Figure S1: Characterization of bone marrow-derived dendritic cells (BMDCs), Figure S2: Human serum media was contained Enpp2. Table S1: Primers used for RT-PCR. Table S2: The counting results of the migration assay in Figure S1D. Table S3: The counting results of the migration assay in Figure 2H. Table S4: The counting results of the migration assay in Figure 3F. Video S1: Homing ability of rmEnpp2-treated DCs in LNs. Video S2: Homing ability of non-treated DCs in LNs.

## References

[1] Gunzer M. (2007). Migration, cell-cell interaction and adhesion in the immune system. Ernst. Scher. Found Symp. Proc..

[2] Platt A.M., Randolph G.J. (2013). Dendritic cell migration through the lymphatic vasculature to lymph nodes. Adv. Immunol..

[3] Takemura S., Braun A., Crowson C., Kurtin P.J., Cofield R.H., O’Fallon W.M., Goronzy J.J., Weyand C.M. (2001). Lymphoid neogenesis in rheumatoid synovitis. J. Immunol..

[4] Hjelmstrom P. (2001). Lymphoid neogenesis: De novo formation of lymphoid tissue in chronic inflammation through expression of homing chemokines. J. Leukoc. Biol..

[5] Biro M., Munoz M.A., Weninger W. (2014). Targeting Rho-GTPases in immune cell migration and inflammation. Br. J. Pharmacol..

[6] Fenteany G., Zhu S. (2003). Small-molecule inhibitors of actin dynamics and cell motility. Curr. Top. Med. Chem..

[7] Luster A.D., Alon R., von Andrian U.H. (2005). Immune cell migration in inflammation: Present and future therapeutic targets. Nat. Immunol..

[8] Mackay C.R. (2008). Moving targets: Cell migration inhibitors as new anti-inflammatory therapies. Nat. Immunol..

[9] Cortinovis M., Aiello S., Mister M., Conde-Knape K., Noris M., Novelli R., Solini S., Rodriguez Ordonez P.Y., Benigni A., Remuzzi G. (2020). Autotaxin Inhibitor Protects from Chronic Allograft Injury in Rat Kidney Allotransplantation. Nephron.

[10] Jankowski M. (2011). Autotaxin: Its role in biology of melanoma cells and as a pharmacological target. Enzym. Res..

[11] Verdijk P., Aarntzen E.H., Punt C.J., de Vries I.J., Figdor C.G. (2008). Maximizing dendritic cell migration in cancer immunotherapy. Expert Opin. Biol. Ther..

[12] Jang M.H., Sougawa N., Tanaka T., Hirata T., Hiroi T., Tohya K., Guo Z., Umemoto E., Ebisuno Y., Yang B.-G. (2006). CCR7 is critically important for migration of dendritic cells in intestinal lamina propria to mesenteric lymph nodes. J. Immunol..

[13] Xu Y., Pektor S., Balkow S., Hemkemeyer S.A., Liu Z., Grobe K., Hanley P.J., Shen L., Bros M., Schmidt T. (2014). Dendritic cell motility and T cell activation requires regulation of Rho-cofilin signaling by the Rho-GTPase activating protein myosin IXb. J. Immunol..

[14] Choo E.H., Lee J.-H., Park E.-H., Park H.E., Jung N.-C., Kim T.-H., Koh Y.-S., Kim E., Seung K.-B., Park C. (2017). Infarcted Myocardium-Primed Dendritic Cells Improve Remodeling and Cardiac Function After Myocardial Infarction by Modulating the Regulatory T Cell and Macrophage Polarization. Circulation.

[15] Grobner S., Lukowski R., Autenrieth I.B., Ruth P. (2014). Lipopolysaccharide induces cell volume increase and migration of dendritic cells. Microbiol. Immunol..

[16] Xiang S.Y., Dusaban S.S., Brown J.H. (2013). Lysophospholipid receptor activation of RhoA and lipid signaling pathways. Biochim. Biophys. Acta.

[17] Miyasaka M., Takeda A., Hata E., Sasaki N., Umemoto E., Jalkanen S. (2016). The Role of Lysophospholipids in Immune Cell Trafficking and Inflammation. Chronic Inflammation.

[18] Van Meeteren L.A., Ruurs P., Stortelers C., Bouwman P., van Rooijen M.A., Pradere J.P., Pettit T.R., Wakelam M.J.O., Saulnier-Blache J.S., Mummery C.L. (2006). Autotaxin, a secreted lysophospholipase D, is essential for blood vessel formation during development. Mol. Cell. Biol..

[19] Tanaka M., Okudaira S., Kishi Y., Ohkawa R., Iseki S., Ota M., Noji S., Yatomi Y., Aoki J., Arai H. (2006). Autotaxin stabilizes blood vessels and is required for embryonic vasculature by producing lysophosphatidic acid. J. Biol. Chem..

[20] Jung N.-C., Lee J.-H., Choi H.-J., Hwang S.-U., Song J.-Y., Seo H.G., Choi J., Jung S.Y., Han S.G., Lim D.-S. (2016). Dendritic Cell Immunotherapy Combined with Cytokine-Induced Killer Cells Effectively Suppresses Established Hepatocellular Carcinomas in Mice. Immunol. Investig..

[21] Ferguson C.G., Bigman C.S., Richardson R.D., van Meeteren L.A., Moolenaar W.H., Prestwich G.D. (2006). Fluorogenic phospholipid substrate to detect lysophospholipase D/autotaxin activity. Org. Lett..

[22] Lee E.G., Jung N.-C., Lee J.-H., Song J.-Y., Ryu S.-Y., Seo H.G., Han S.G., Ahn K.J., Hong K.S., Choi J. (2016). Tolerogenic dendritic cells show gene expression profiles that are different from those of immunogenic dendritic cells in DBA/1 mice. Autoimmunity.

[23] Palucka K., Banchereau J. (2012). Cancer immunotherapy via dendritic cells. Nat. Rev. Cancer.

[24] Garg A.D., Coulie P.G., Van den Eynde B.J., Agostinis P. (2017). Integrating Next-Generation Dendritic Cell Vaccines into the Current Cancer Immunotherapy Landscape. Trends Immunol..

[25] Kelley N., Jeltema D., Duan Y., He Y. (2019). The NLRP3 Inflammasome: An Overview of Mechanisms of Activation and Regulation. Int. J. Mol. Sci..

[26] Savaskan N.E., Rocha L., Kotter M., Baer A., Lubec G., van Meeteren L., Kishi Y., Aoki J., Moolenaar W.H., Nitsch R. (2007). Autotaxin (NPP-2) in the brain: Cell type-specific expression and regulation during development and after neurotrauma. Cell. Mol. Life Sci..

[27] Brisbin A.G., Asmann Y.W., Song H., Tsai Y.-Y., A Aakre J., Yang P., Jenkins R.B., Pharoah P., Schumacher F., Conti D.V. (2011). Meta-analysis of 8q24 for seven cancers reveals a locus between NOV and ENPP2 associated with cancer development. BMC Med. Genet..

[28] Chen M., O’Connor K.L. (2005). Integrin alpha6beta4 promotes expression of autotaxin/ENPP2 autocrine motility factor in breast carcinoma cells. Oncogene.

[29] D’Souza K., Nzirorera C., Cowie A.M., Varghese G.P., Trivedi P., Eichmann T.O., Biswas D., Touaibia M., Morris A.J., Aidinis V. (2018). Autotaxin-LPA signaling contributes to obesity-induced insulin resistance in muscle and impairs mitochondrial metabolism. J. Lipid Res..

[30] Knowlden S., Georas S.N. (2014). The autotaxin-LPA axis emerges as a novel regulator of lymphocyte homing and inflammation. J. Immunol..

[31] Randolph G.J., Ochando J., Partida-Sanchez S. (2008). Migration of dendritic cell subsets and their precursors. Annu. Rev. Immunol..

[32] Bros M., Haas K., Moll L., Grabbe S. (2019). RhoA as a Key Regulator of Innate and Adaptive Immunity. Cells.

[33] Panther E., Idzko M., Corinti S., Ferrari D., Herouy Y., Mockenhaupt M., Dichmann S., Gebicke-Haerter P., Di Virgilio F., Girolomoni G. (2002). The Influence of Lysophosphatidic Acid on the Functions of Human Dendritic Cells. J. Immunol..

[34] Chen R., Roman J., Guo J., West E., McDyer J., Williams M.A., Georas S.N. (2006). Lysophosphatidic Acid Modulates the Activation of Human Monocyte-Derived Dendritic Cells. Stem Cells Dev..

[35] Chan L.C., Peters W., Xu Y., Chun J., Farese R.V., Caseschen S. (2007). LPA3 receptor mediates chemotaxis of immature murine dendritic cells to unsaturated lysophosphatidic acid (LPA). J. Leukoc. Biol..

[36] Salous A.K., Panchatcharam M., Sunkara M., Mueller P., Dong A., Wang Y., Graf G.A., Smyth S.S., Morris A.J. (2013). Mechanism of rapid elimination of lysophosphatidic acid and related lipids from the circulation of mice. J. Lipid Res..

[37] Albers H.M.H.G., Dong A., van Meeteren L.A., Egan D.A., Sunkara M., van Tilburg E.W., Schuurman K., van Tellingen O., Morris A.J., Smyth S.S. (2010). Boronic acid-based inhibitor of autotaxin reveals rapid turnover of LPA in the circulation. Proc. Natl. Acad. Sci. USA.

[38] Ridley A.J. (2015). Rho GTPase signalling in cell migration. Curr. Opin. Cell. Biol..

[39] Katakai T., Kondo N., Ueda Y., Kinashi T. (2014). Autotaxin produced by stromal cells promotes LFA-1-independent and Rho-dependent interstitial T cell motility in the lymph node paracortex. J. Immunol..

[40] Lu Y., Zhang M., Wang S., Hong B., Wang Z., Li H., Zheng Y., Yang J., Davis R.E., Qian J. (2014). p38 MAPK-inhibited dendritic cells induce superior antitumour immune responses and overcome regulatory T-cell-mediated immunosuppression. Nat. Commun..

[41] Fessler M.B., Arndt P.G., Just I., Nick J.A., Malcolm K.C., Worthen G.S. (2007). Dual role for RhoA in suppression and induction of cytokines in the human neutrophil. Blood.

[42] Martino A., Volpe E., Baldini P.M. (2006). The influence of lysophosphatidic acid on the immunophenotypic differentiation of human monocytes into dendritic cells. Haematologica.

[43] Clatworthy M.R., Aronin C.E., Mathews R.J., Morgan N.Y., Smith K.G., Germain R.N. (2014). Immune complexes stimulate CCR7-dependent dendritic cell migration to lymph nodes. Nat. Med..

