# Supplementary material for: Enpp2 Expression by Dendritic Cells Is a Key Regulator in Migration
Source: Biomedicines. 2021 Nov 19;9(11):1727. doi: 10.3390/biomedicines9111727 (PMC8615729; doi:10.3390/biomedicines9111727)
Supplement: Supplementary file 1 [file biomedicines-09-01727-s001.zip › Supplemental DATA.pdf]

Supplemental DATA

Methods

Western blotting

To detect Enpp2, RPMI/3% human AB serum (R/HS) and serum free medium (CellGro; CellGenix, Freiburg, Germany and X-VIVO15; Lonza, Basel, Switzerland) were concentrated by ultracentrifugation. Equal amounts of protein (200 μg) were separated on SDS-PAGE gels and transferred to PVDF membranes. The membranes were blocked with 5% (w/w) skim milk in TBST and then incubated with primary antibodies specific for ENPP2 (Diluted 1:200; Cayman Chemical, Ann Arbor, MI, USA). The membranes were then incubated with an HRP-conjugated anti-rabbit antibody (Abcam, Cambridge, MA, UK). The membranes were exposed to ECL reagents (Thermo Scientific) and signals were detected using a Luminescent image analyzer (LAS-4000; Fujifilm, Tokyo, Japan).

Migration assay and in vivo tracking

For visualization and imaging, DCs were labeled with carboxyfluorescein succinimidyl ester (CFSE) (Invitrogen). Cells (5×10^3 rmEnpp2-treated DCs or non-treated DCs) were injected into the footpad of C57BL/6 mice. At 3 days post-injection of CFSE-labeled DCs, mice were sacrificed and popliteal LNs were harvested. CFSE signals (green) and nuclei (DAPI; blue) in LNs were visualized under a confocal microscope (Carl Zeiss, USA). Videos were obtained by combining distinct sequential images (z axis projection merge of the 20 plane images) using ImageJ software. Images were obtained by optical sectioning at 10 μm increments from the outer layer to the core along the Z-axis of a popliteal lymph nodes. All images were taken using the same microscope settings and representative images are shown.

Table S1. Primers used for RT-PCR.

Name	Direction	Sequence							
mEnpp2	Forward	GTC	CTC	TCT	CTG	TGT	CTT	CTT	TC
	Reverse	CAT	GAG	TTC	CTC	TAC	CCA	CTT	C
hEnpp2	Forward	GAA	CAT	CAT	CCC	TGC	CTC	TA	
	Reverse	CCT	GCT	TCA	CCA	CCT	TCT	T	
Lpar1	Forward	CAA	ACG	CTG	GAG	GAG	ATG	AA	
	Reverse	GGG	AAT	GGA	GCC	CAA	GTA	AA	
Lpar2	Forward	TTC	AGG	GAG	GCT	CTG	TAT	CT	
	Reverse	CTT	TCA	GGA	CTT	GGG	TCT	CTA	C
Lpar3	Forward	CTC	TTC	TCT	GGC	TCC	CAT	TTA	C
	Reverse	CAC	TGC	TCA	TCT	CTC	CCT	TTC	
Lpar4	Forward	CCT	AGT	CCT	CAG	TGG	TGG	TAT	T
	Reverse	GAT	AGC	TGG	AAG	GGA	AGG	TTT	G
Lpar5	Forward	CTG	TGC	TTC	GTG	CCC	TAT	AA	
	Reverse	CTG	GAG	TAG	AAC	CTG	ACT	TGT	G
Lpar6	Forward	CGT	TTG	CAT	TGC	TGT	GTG	GTT	C
	Reverse	GGC	CGC	TGG	AAA	GTT	CTC	AAA	G
mGAPDH	Forward	AAC	AGC	AAC	TCC	CAC	TCT	TC	
	Reverse	CCT	GTT	GCT	GTA	GCC	GTA	TT	
hGAPDH	Forward	TCA	GAG	GAC	GAA	TCA	AAA	TGG	G
	Reverse	CAG	GTA	TGT	CTT	GAG	TGT	CAG	G
mCCR7	Forward	GCT	GCG	TCA	ACC	CTT	TCT	TG	
	Reverse	ACC	GAC	GCG	TTC	CGT	ACA	T	

Table S2. The counting results of the migration assay in Fig S1D.

Counts	Fig S1D	
	imDC	mDC
1	8,145 cells	13,815 cells
2	8,010 cells	13,654 cells
3	7,980 cells	13,210 cells

Table S3. The counting results of the migration assay in Fig 2H.

Counts	Fig 2H	
	SC	siEnpp2
1	5,340 cells	4,440 cells
2	5,520 cells	4,305 cells
3	5,205 cells	4,155 cells

Table S4. The counting results of the migration assay in Fig 3F.

Counts	Fig 3F	
	Enpp2-	Enpp2+
1	4,470 cells	5,775 cells
2	4,380 cells	6,120 cells
3	4,530 cells	5,910 cells

Figures (with legends)

Supplementary Figure 1.

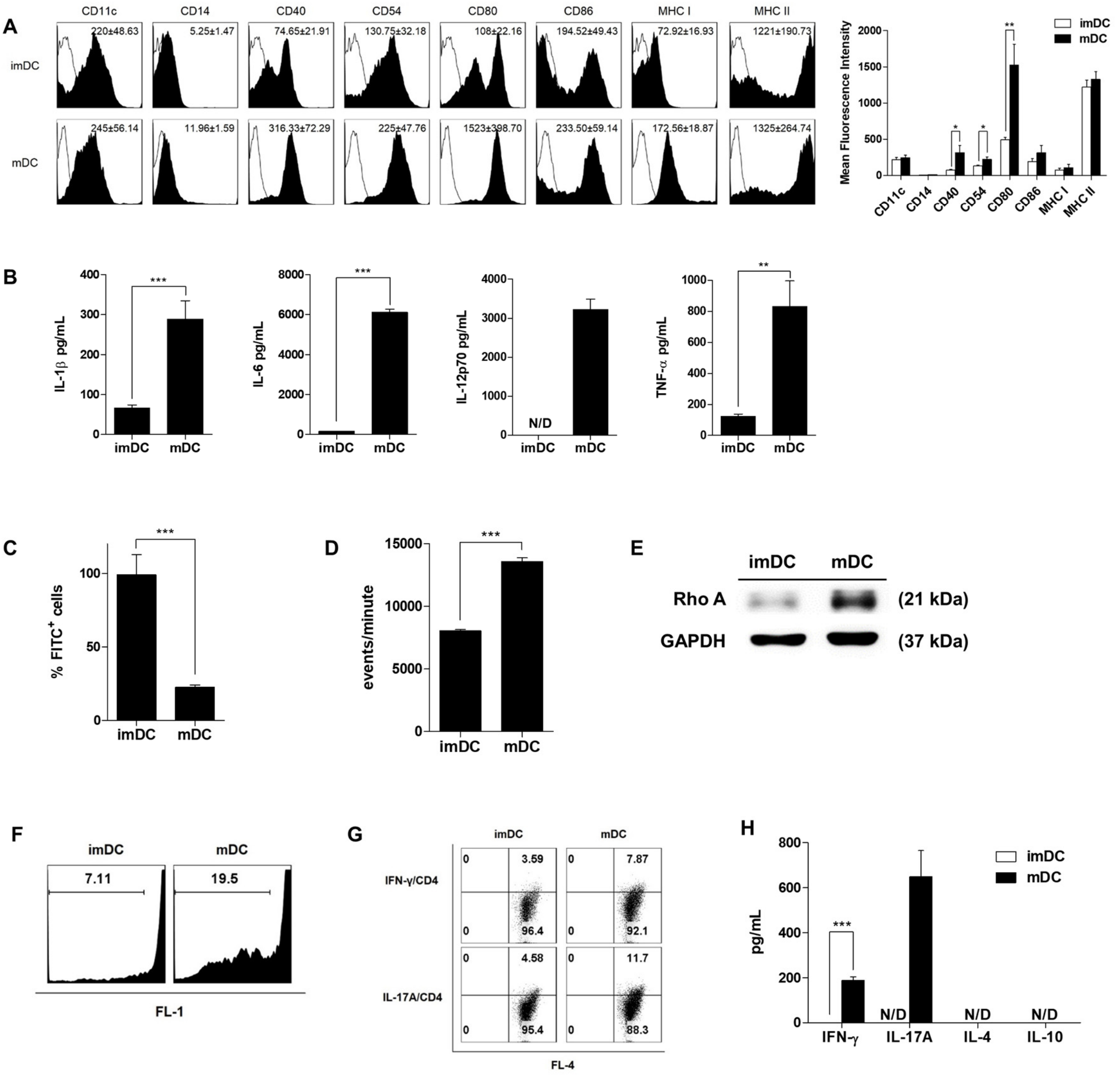

Supplemental Figure S1. Characterization of bone marrow-derived dendritic cells (BMDCs).

(A) DC subsets (imDCs and mDCs) were stained with fluorescently conjugated antibodies specific for the indicated molecules and analyzed by flow cytometry. The histograms are representative of five independent DC preparations. The bar graphs show the mean fluorescence intensity (MFI), expressed as the mean ± SEM (n = 5 independent DC

preparations). (B) Pro-inflammatory cytokines in the culture supernatants of DC subsets were analyzed by ELISA. Data are expressed as the mean ± SEM (n = 5 independent DC preparations) of duplicate experiments. (C) imDCs and mDCs were prepared and their ability to uptake FITC-conjugated dextran was compared. Each DC subset (2×10^5 cells/mL) was incubated for 1 h with 1.0 mg/mL FITC-dextran. The percentage of FITC-dextran-positive cells was determined by flow cytometry. The data in the bar graphs are expressed as delta MFI (ΔMFI), which was calculated by subtracting the MFI value for non-specific FITC-dextran uptake at 4°C from the MFI values obtained at 37°C. The bar graph shows the MFI, expressed as the mean ± SEM (n = 5 independent DC preparations). (D) Migration assay. The number of migrating DCs harvested from the lower Transwell chamber was counted by flow cytometry. The bar graph shows the events/minute, expressed as the mean ± SEM (n = 3 independent DC preparations). (E) DC subsets were cultured for 7 days and cell lysates were subjected to SDS-PAGE and im-munoblotting to detect Rho A protein. (F) Each DC subsets was co-cultured for 72 h with CD3+ T cells at a ratio of 1:10 to measure T cell proliferation. $CD3^+$ T cells isolated from splenocytes of naïve C57BL/6 mice were stained with carboxyfluorescein succinimidyl ester (CFSE) at a final concentration of 4 μM. CFSE-labeled cells were washed, counted, and co-cultured with DCs. (G) Each DC subset was co-cultured for 72 h with $CD3^+$ T cells at a ratio of 1:10. To identify Th1 and Th17 populations, cells were stained intracellularly with anti-CD4, anti-IFN-γ, or anti-IL-17A antibodies and then analyzed by flow cytometry. (H) Cytokine levels in the supernatants after 72 h of co-culture, as measured by ELISA. Data are expressed as the mean ± SEM (n = 5 independent co-culture preparations). *$P < 0.05$, **$P < 0.01$, and ***$P < 0.001$, compared with imDCs.

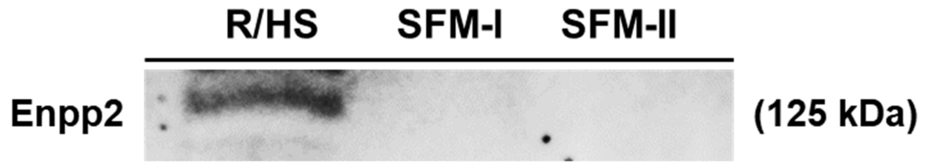

Supplemental Figure S2. Human serum media was contained Enpp2.

Levels of Enpp2 expression in RPMI/3% human serum media (R/HS) and two types of serum free media (SFM-I; CellGro, SFM-II; X-VIVO) was detected by western blotting. R/HS contained Enpp2 but SFMs did not.

Legends for videos

Supplemental video S1. Homing ability of rmEnpp2-treated DCs in LNs.

rmEnpp2-treated DCs (green) and nuclei (DAPI; blue) in LNs were visualized under a confocal microscope. Scale bar, 50 μm.

Supplemental video S2. Homing ability of non-treated DCs in LNs.

Non-treated DCs (green) and nuclei (DAPI; blue) in LNs were visualized under a confocal microscope. Scale bar, 50 μm.
